# Amyloid β causes excitation/inhibition imbalance through dopamine receptor 1-dependent disruption of fast-spiking GABAergic input in anterior cingulate cortex

**DOI:** 10.1038/s41598-017-18729-5

**Published:** 2018-01-10

**Authors:** Si-Qiang Ren, Wen Yao, Jing-Zhi Yan, Chunhui Jin, Jia-Jun Yin, Jianmin Yuan, Shui Yu, Zaohuo Cheng

**Affiliations:** 1 Wuxi Mental Health Center, Nanjing Medical University, Wuxi, China; 2 Department of Pharmacology, Wuxi Higher Health Vocational Technology School, Wuxi, China; 3 Jiangsu Key Laboratory of Brain Disease Bioinformation, Research Center for Biochemistry and Molecular Biology, Xuzhou Medical College, Xuzhou, China

## Abstract

Alzheimer’s disease (AD) is the most common cause of dementia in the elderly. At the early stages of AD development, the soluble β-amyloid (Aβ) induces synaptic dysfunction, perturbs the excitation/inhibition balance of neural circuitries, and in turn alters the normal neural network activity leading to cognitive decline, but the underlying mechanisms are not well established. Here by using whole-cell recordings in acute mouse brain slices, we found that 50 nM Aβ induces hyperexcitability of excitatory pyramidal cells in the cingulate cortex, one of the most vulnerable areas in AD, via depressing inhibitory synaptic transmission. Furthermore, by simultaneously recording multiple cells, we discovered that the inhibitory innervation of pyramidal cells from fast-spiking (FS) interneurons instead of non-FS interneurons is dramatically disrupted by Aβ, and perturbation of the presynaptic inhibitory neurotransmitter gamma-aminobutyric acid (GABA) release underlies this inhibitory input disruption. Finally, we identified the increased dopamine action on dopamine D1 receptor of FS interneurons as a key pathological factor that contributes to GABAergic input perturbation and excitation/inhibition imbalance caused by Aβ. Thus, we conclude that the dopamine receptor 1-dependent disruption of FS GABAergic inhibitory input plays a critical role in Aβ-induced excitation/inhibition imbalance in anterior cingulate cortex.

## Introduction

Alzheimer’s disease (AD), the leading cause of dementia in the elderly, is characterized by pathological hallmark of extracellular Amyloid β deposits^1^. However, it is becoming increasingly clear that at early preclinical stages even before amyloid is deposited, the accumulated soluble Aβ disrupts synaptic transmission, perturbs excitation/inhibition (E/I) balance and alters neuronal networks resulting in the cognitive decline in AD^2–4^. AD patients with early-onset dementia have an increased risk of epileptic seizures^5,6^. Consistently, AD model mice that overexpress Aβ also show hyperexcitation in individual neurons and higher epileptiform activity in cortical and hippocampal networks^7–11^. Similarly, Aβ at pathological relevant concentrations cause neuronal hyperexcitation in culture neurons^9,11–14^. Hence, Aβ-induced neuronal hyperexcitation and epilepsy are believed to represent the excitotoxic effect which leads to neuronal silencing and cognitive deficits^8,15,16^. However, it is still not well established how Aβ induces neuronal hyperexcitation.

Anterior cingulate cortex (ACC) is a part of the medial prefrontal cortex, which plays a pivotal role in memory, attention and emotion^17–19^. Dysfunction of ACC metabolism and functional connectivity are involved in aging-related cognitive decline^20–22^. ACC is one of the earliest affected areas and “epicenters” in AD^23–26^. ACC is also one of the most selective areas where Aβ accumulates at the very early stage in AD patients^23^. However, how Aβ influences the local circuits in ACC is elusive.

In ACC, the proper GABAergic inhibitory innervation of excitatory pyramidal cells is important for spatial and temporal dynamics in cognitive processes. Disruption of excitation/inhibition balance is related to many psychiatric diseases such as schizophrenia, epilepsy and autism^27–30^. Inhibitory interneurons can be classified as fast-spiking (FS) and non-FS cells based on their firing patterns^31^. FS interneuron is the predominant subtype in mammalian neocortex, and it primarily innervates the soma and the axonal initial segment of excitatory pyramidal cells to control action potential (AP) firing and synchronization, whereas non-FS interneurons preferentially target dendrites to control efficacy and plasticity of excitatory inputs^32–34^. Interestingly in the frontal cortex, the inhibitory innervation of pyramidal cells from FS and non-FS interneurons can be regulated differently by the enriched dopaminergic input from areas such as the ventral tegmental area^35,36^. Abnormal dopaminergic innervation of FS parvalbumin interneurons has been suggested to exaggerate schizophrenia symptom by disrupting E/I balance^37^. In AD, Aβ promotes excessive dopamine release in the frontal cortex^38^, and dopamine receptor 1 (D1 receptor) is involved in Aβ-induced epileptic activity^39^. Nevertheless, whether the dopamine-related signaling pathway is directly involved in Aβ-induced neuronal hyperexcitation has not yet been studied.

Here by using whole-cell recordings in acute mouse brain slices, we found that 50 nM Aβ leads to hyperexcitability of excitatory pyramidal cells in ACC through specifically depressing inhibitory synaptic innervation from FS but not non-FS interneurons. We also discovered that perturbation of presynaptic GABA release is the main cause of this inhibitory input disruption. In addition, we identified that the excessive activation of dopamine D1 receptor of FS interneurons leads to Aβ-induced disruption of inhibitory innervation. More importantly, D1 receptor antagonist SCH23390 can reverse Aβ-induced hyperexcitability of pyramidal cells. This suggests that the increased dopamine action on D1 receptor of FS interneurons is the key mechanism in this pathological process.

## Results

### 50 nM Aβ promotes pyramidal cell excitability in ACC

**Figure 1:**
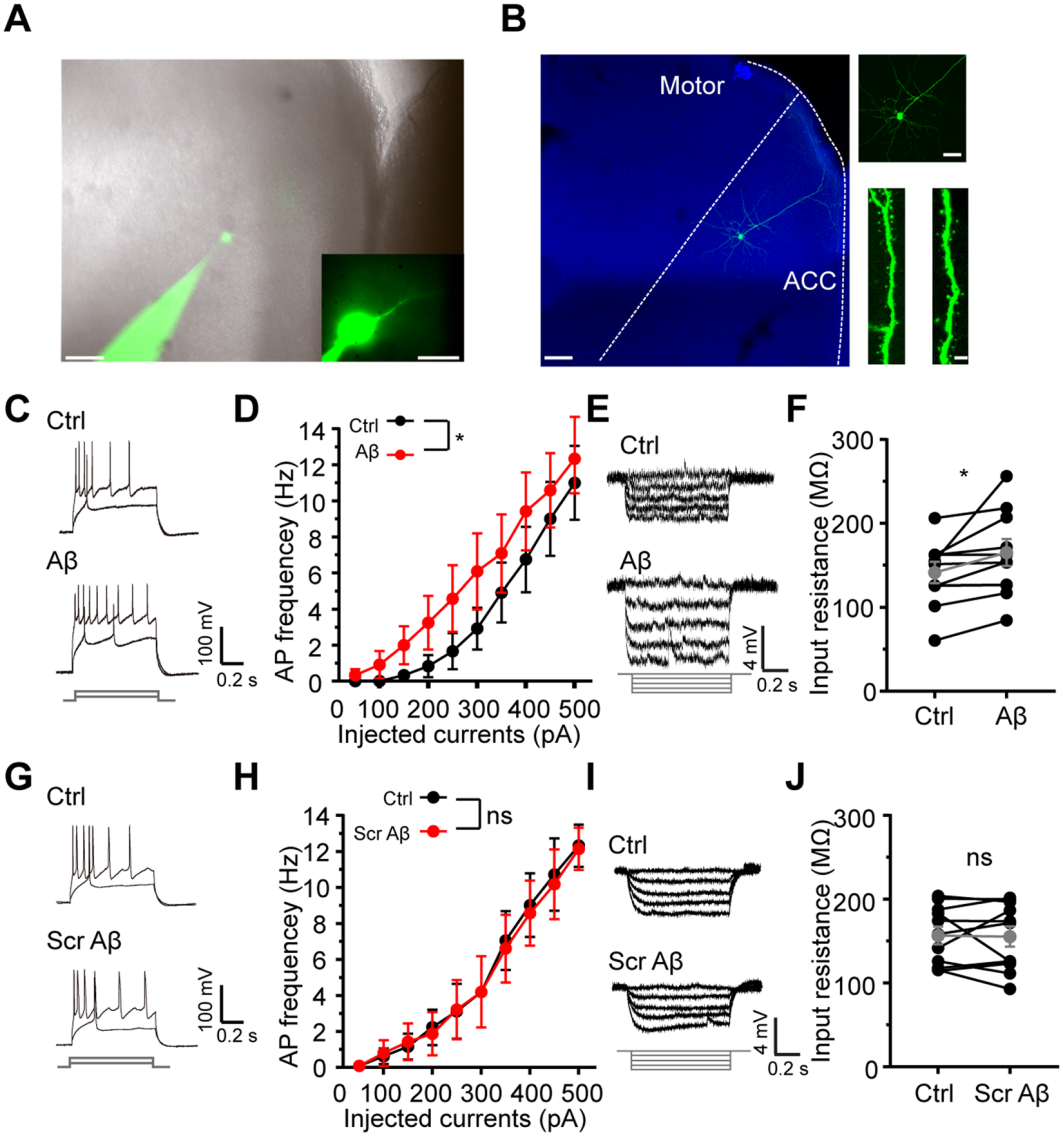
Aβ induces hyperexcitability of excitatory pyramidal cells in ACC. (**A**) DIC epifluorescent image of a pyramidal cell in whole-cell configuration in ACC. Alexa 488-conjugated biocytin (green, left) was included in the recording pipette to reveal cell morphology and confirm the cells identity. Scale bars: 100 µm and 20 µm; (**B**) high-resolution confocal scanning image of the pyramidal cell in (**A**) (left); zoom-in images showed typical pyramidal shape soma (right, top) and apical and basal dendrites and spines (right, down). Scale bars: 100 µm, 50 µm and 5 µm; (**C**) examples of AP traces induced by 150 pA and 400 pA current injections before and after Aβ application; (**D**) quantification of the AP frequencies at different current injections before and after Aβ application; (**E**) examples of membrane potential responses induced by serial currents injection from −20 pA to 0 pA with 5 pA interval before and after Aβ application; (**F**) quantification of cellular input resistance before and after Aβ application; (**G**) examples of AP traces induced by 150 pA and 400 pA current injections before and after Scr-Aβ application; (**H**) quantification of the AP frequencies at different current injections before and after Scr-Aβ application; (**I**) examples of membrane potential responses induced by serial currents injection from −20 pA to 0 pA before and after Aβ application; (**F**) quantification of cellular input resistance before and after Scr-Aβ application. (*p < 0.05).

Whole cell recordings, as previously reported^40,41^, were performed on ACC excitatory pyramidal cells in acute brain slices (Fig. 1A,B). Confocal images and AP firing patterns confirmed the identity of excitatory pyramidal cells (Fig. 1B,C,G). To evaluate the effects of Aβ on neuronal excitability, a series of depolarizing currents were injected to elicit APs before (Ctrl) and 5–10 mins after 50 nM Aβ administration (Aβ) into perfusing artifical cerebrospinal fluid (ACSF). The frequency of APs was dramatically increased after Aβ administration (p = 0.0251, compared with Ctrl, repeated-measures two-way ANOVA, n = 10 cells; Fig. 1C,D). Moreover, the input resistance was also significantly increased after Aβ administration (Aβ: 164.08 ± 16.50 MΩ, n = 10 cells, p = 0.0372 compared with Ctrl: 145.83 ± 12.30 MΩ, paired *t* test; Fig. 1E,F). At the same time, Aβ also increased the frequency but not the amplitude of spontaneous postsynaptic excitatory currents (sp EPSCs) (frequency: Aβ: 4.51 ± 0.90 Hz, n = 8 cells, p = 0.0042 compared with Ctrl: 3.86 ± 0.87 Hz; amplitude: Aβ: 6.56 ± 0.65 pA, n = 8 cells, p = 0.3830 compared with Ctrl: 6.06 ± 0.60 pA; paired *t* test; Supplemental Fig. 1A,B). As a control, the scrambled Aβ (Scr-Aβ) had no any affect on the AP firing frequency (p = 0.6532, compared with Ctrl, repeated-measures two-way ANOVA, n = 14 cells; Fig. 1G,H), input resistance (Scr-Aβ: 155.35 ± 12.08 MΩ, n = 11 cells, p = 0.8724 compared with Ctrl: 156.63 ± 10.60 MΩ, paired *t* test; Fig. 1I,J) and sp EPSCs (frequency: Scr-Aβ: 3.33 ± 0.80 Hz, n = 8 cells, p = 0.4592 compared with Ctrl: 3.42 ± 0.73 Hz; amplitude: Scr-Aβ: 6.72 ± 0.93 pA, n = 8 cells, p = compared with Ctrl: 6.73 ± 0.82 pA; paired *t* test; Supplemental Fig. 1C,D). These results strongly indicate that Aβ promotes pyramidal cell excitation in ACC. Interestingly, this increased neuronal excitation caused by Aβ exposure was reversed 30 mins after Aβ washout (Washout: p = 0.0287 compared with Aβ, n = 5 cells; p = 0.1709 compared with Ctrl, n = 5 cells; p = 0.0105, Aβ compared with Ctrl; repeated-measures two-way ANOVA; Supplemental Fig. 2A,B), implying Aβ-induced neuronal hyperexcitability in ACC pyramidal cells is an acute effect. Taken together, all the results demonstrate Aβ exposure can promote neuronal excitation of excitatory pyramidal cells in ACC.

### Aβ promotes pyramidal cell excitability by disrupting presynaptic inhibitory input

**Figure 2:**
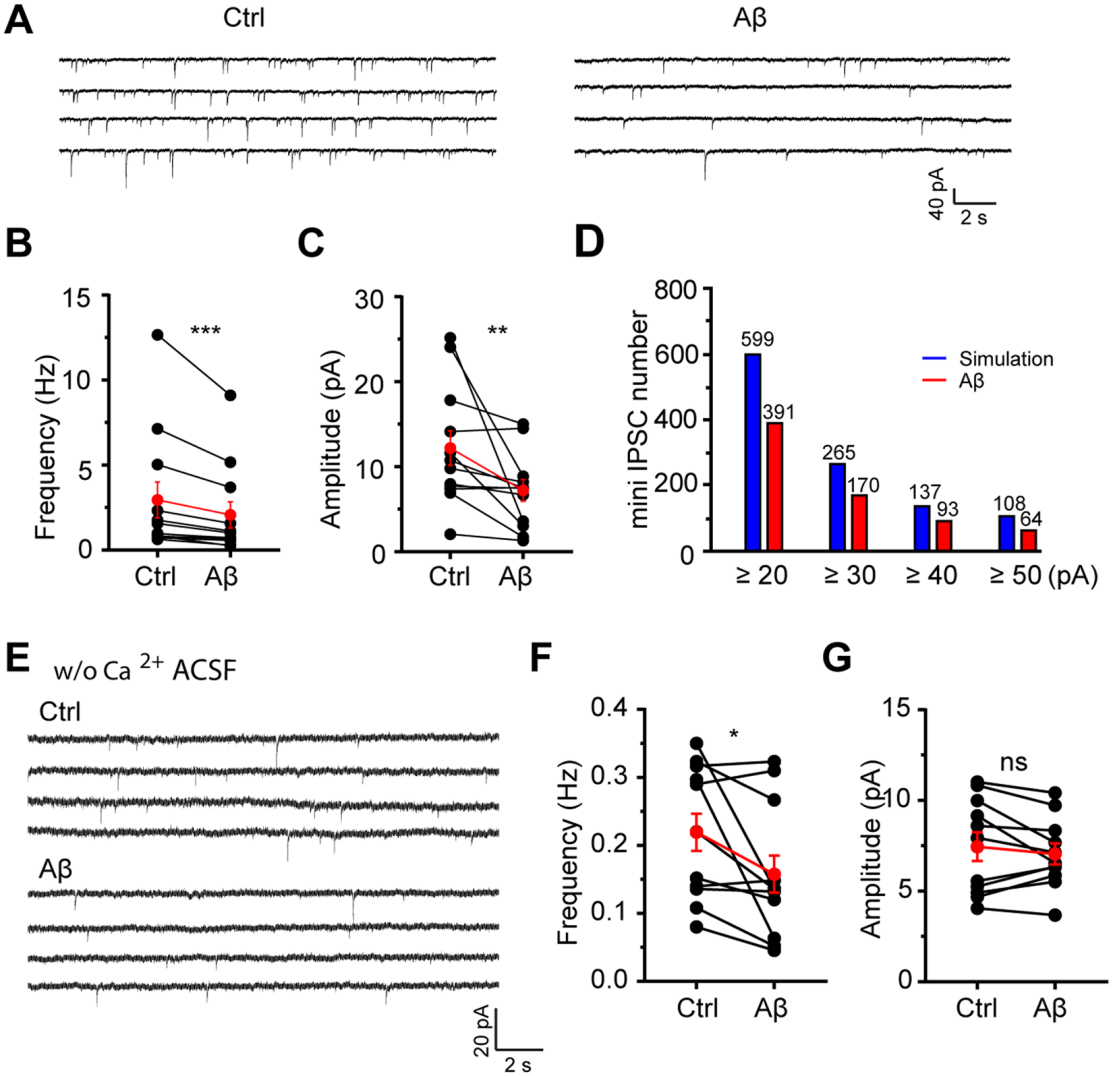
Aβ disrupts inhibitory input selectively by perturbing presynaptic GABA release in ACC. (**A**) Example traces of mini IPSCs of excitatory pyramidal cells in ACC before and after Aβ application; (**B**) quantification of mini IPSCs frequency before and after Aβ application; (**C**) quantification of mini IPSCs amplitude before and after Aβ application; (**D**) numbers of large-amplitude mini IPSCs in the simulated and Aβ conditions. The simulation was done based on the decreased percentages of mini IPSCs frequency and amplitude shown in (**B**) and (**C**); (**E**) example traces of mini IPSCs of excitatory pyramidal cells in ACC before and after Aβ application recorded in calcium-free perfusing ACSF; (**F**) quantification of mini IPSCs frequency before and after Aβ application in calcium-free perfusing ACSF; (**G**) quantification of mini IPSCs amplitude before and after Aβ application in calcium-free perfusing ACSF; Wilcoxon signed-rank test (*p < 0.05;**p < 0.01, ***p < 0.001).

To assess if the inhibitory input disruption is related to the enhanced excitation of pyramidal cells caused by Aβ in ACC, miniature inhibitory postsynaptic currents (mini IPSCs) were recorded from pyramidal cells using high-chloride internal solution with TTX and NBQX incubated in the perfusing ACSF (Supplemental Fig. 3). Aβ caused a dramatic decrease of both frequency (Aβ: 2.07 ± 0.77 Hz, n = 12 cells, p = 0.0005 compared with ctrl: 2.95 ± 1.06 Hz, Wilcoxon signed-rank test; Fig. 2A,B) and amplitude (Aβ: 7.13 ± 1.27 pA, n = 12 cells, p = 0.0034 compared with ctrl: 12.12 ± 2.03 pA; Wilcoxon signed-rank test; Fig. 2A,C) of mini IPSCs. This indicates that the enhanced excitation of pyramidal cells during Aβ exposure is possibly attributed to inhibitory input disruption.

The decreased frequency of mini IPSCs is exclusively due to the perturbed presynaptic GABA release, but the reduced amplitude could be resulted from a decrease in either postsynaptic GABA receptor number or presynaptic multi-vesicular events^42^. We assumed that the reduced postsynaptic GABA receptor number decreases the amplitudes of all mini IPSCs uniformly, whereas the reduced presynaptic multi-vesicular events decreases the number of large-amplitude mini IPSCs selectively because the large-amplitude mini IPSCs are mostly resulted from multi-vesicular events^42^. In order to figure out which mechanism is the main cause of the overall decreased mini IPSCs amplitude, we simulated the amplitude distribution of mini IPSCs (Ctrl: 7595 events; Aβ: 5939 events) based on Fig. 2B,C, the frequency decreased 30% (from 2.97 Hz to 2.07 Hz) and the amplitude decreased 42% (from 12.12 pA to 7.13 pA) uniformly for all the mini IPSCs. Surprisingly, the number of large-amplitude mini IPSCs after Aβ administration was dramatically fewer compared to the simulated number (Fig. 2D), hinting the overall decreased mini IPSCs amplitude in Fig. 2A,C is probably due to the reduced presynaptic multi-vesicular events.

To directly prove that the presynaptic GABA release disruption is the main mechanism underlying Aβ-induced inhibitory input disruption, we recorded mini IPSCs in calcium-free perfusing ACSF as a lowered external calcium concentration can eliminate multi-vesicular events^42^. In calcium-free perfusing fluid, Aβ caused a significant decrease of the frequency (Aβ: 0.16 ± 0.03 Hz, n = 10 cells, p = 0.0322 compared with ctrl: 0.22 ± 0.03 Hz; Wilcoxon signed-rank test; Fig. 2E,F) but not amplitude (Aβ: 7.14 ± 0.59 pA, n = 10 cells, p = 0.4131 compared with ctrl: 7.94 ± 0.95 pA; Wilcoxon signed-rank test; Fig. 2E,G) of mini IPSCs. This suggests the impairment of presynaptic GABA release is the main mechanism underlying Aβ-induced inhibitory input disruption.

To determine whether disruption of GABAergic inhibitory input plays a causal role in the increased excitation of pyramidal cells induced by Aβ, GABA_A_ receptor antagonist bicuculline methiodide (BMI) was applied together with Aβ. With GABA_A_ receptors blocked by BMI, the frequency of APs evoked by serial currents injection (p = 0.5344 compared with Ctrl, repeated-measures two-way ANOVA, n = 12 cells; Supplemental Fig. 4A,B) and the input resistance (203.89 ± 19.64 MΩ, n = 11 cells, p = 0.2869 compared with ctrl: 210.57 ± 19.55 MΩ; paired *t* test; Supplemental Fig. 4A,B) were not changed after Aβ administration, suggesting GABAergic inhibitory input is involved in the Aβ-induced hyperexcitability of pyramidal cells in ACC.

Taken together, all the above results indicate that Aβ promotes pyramidal cell excitation by disrupting presynaptic GABAergic inhibitory input.

### Inhibitory input from FS interneurons is preferentially disrupted by Aβ

**Figure 3:**
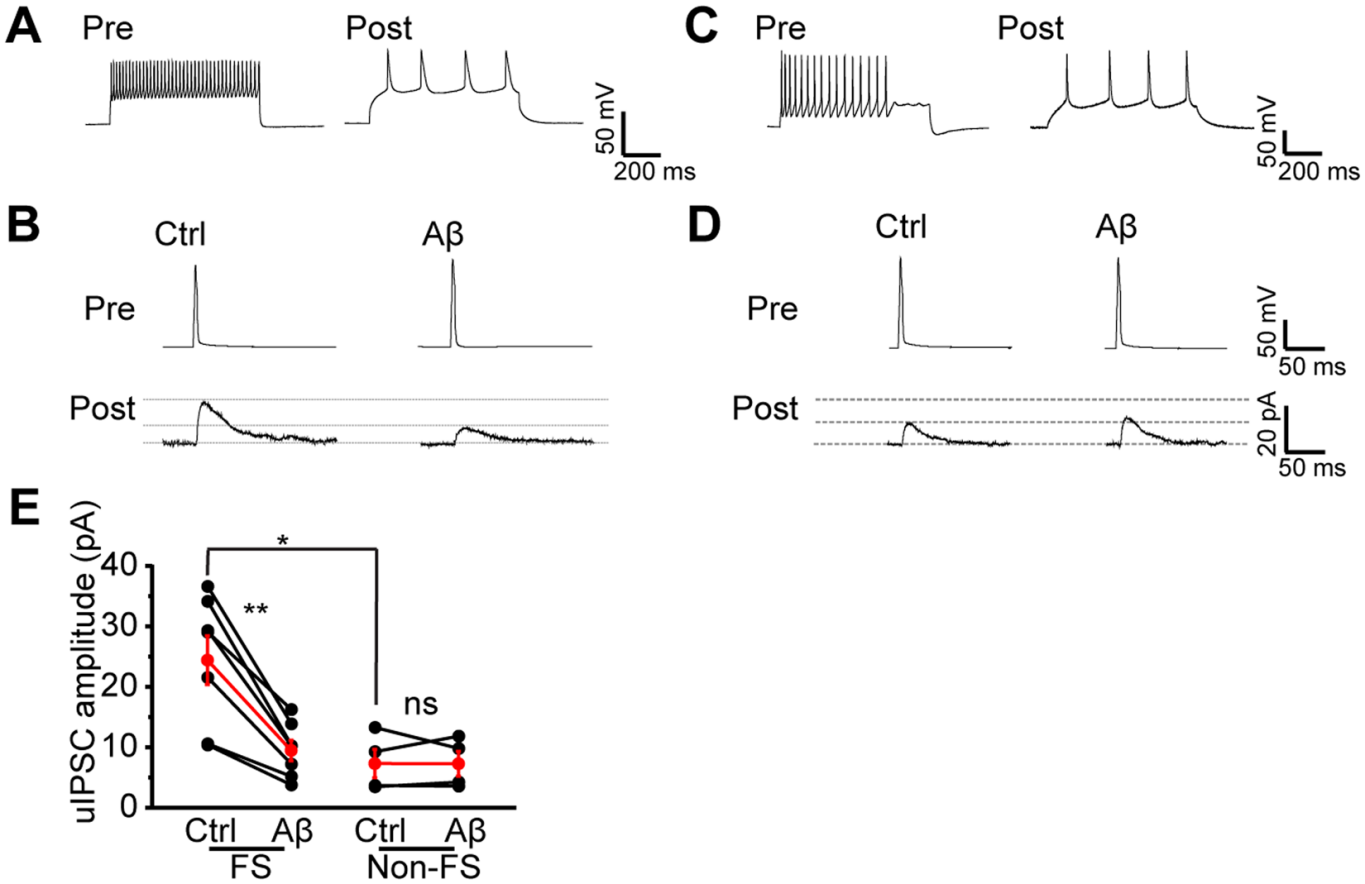
Aβ disrupts inhibitory synaptic connection from FS but not non-FS interneurons to pyramidal cells. (**A**) Example AP traces of a pair of nearby FS interneuron and excitatory neuron simultaneously recorded; (**B**) example traces of uIPSCs triggered by brief current injection to the FS interneuron and recorded in the pyramidal neuron before and after Aβ application; (**C**) example AP traces of a pair of nearby non-FS interneuron and excitatory neuron simultaneously recorded; (**D**) example traces of uIPSCs triggered by brief current injection to the non-FS interneuron and recorded in the pyramidal neuron before and after Aβ application; (**E**) quantification of uIPSCs amplitude from FS and non-FS interneurons before and after Aβ application. (*p < 0.05, **p < 0.01).

The predominant interneuron subtype in the neocortex is the FS interneurons, which preferentially targets the soma and the axonal initial segment of pyramidal cells to control AP output and synchronization^32–34^. In contrast, non-FS interneurons primarily target the dendrites to control the efficacy and plasticity of excitatory inputs onto pyramidal cells^32–34^. To test if Aβ uniformly disrupts inhibitory inputs from both FS and non-FS interneurons, we simultaneously recorded interneurons and pyramidal cells to specifically detect unitary inhibitory postsynaptic currents (uIPSCs) from either FS or non-FS interneurons to pyramidal cells. A 500 ms current was injected into cells to determine the cell types (Fig. 3A,C). A brief current was injected into interneuron to trigger single AP and evoke uIPSCs in pyramidal cells. As previously reported^43^, the amplitude of uIPSCs from FS interneurons to pyramidal cells was more robust than that from non-FS interneurons to pyramidal cells (FS: 24.53 ± 4.04 pA, n = 7 pairs of cells, p = 0.015 compared with non-FS: 7.43 ± 2.37 pA, n = 4 pairs of cells, independent *t* test; Fig. 3B,D,E). Aβ application caused a dramatic decrease of uIPSCs amplitude from FS interneurons (Aβ: 9.57 ± 1.71 pA, n = 7 pairs of cells, p = 0.002 compared with Ctrl: 24.53 ± 4.04 pA, paired *t* test; Fig. 3B,E) but not non-FS interneurons (Aβ: 7.37 ± 2.04 pA, n = 4 pairs of cells, p = 0.969 compared with Ctrl: 7.43 ± 2.37 pA, paired *t* test; Fig. 3C,E), suggesting that GABAergic inhibitory input from FS interneurons is preferentially disrupted by Aβ.

**Figure 4:**
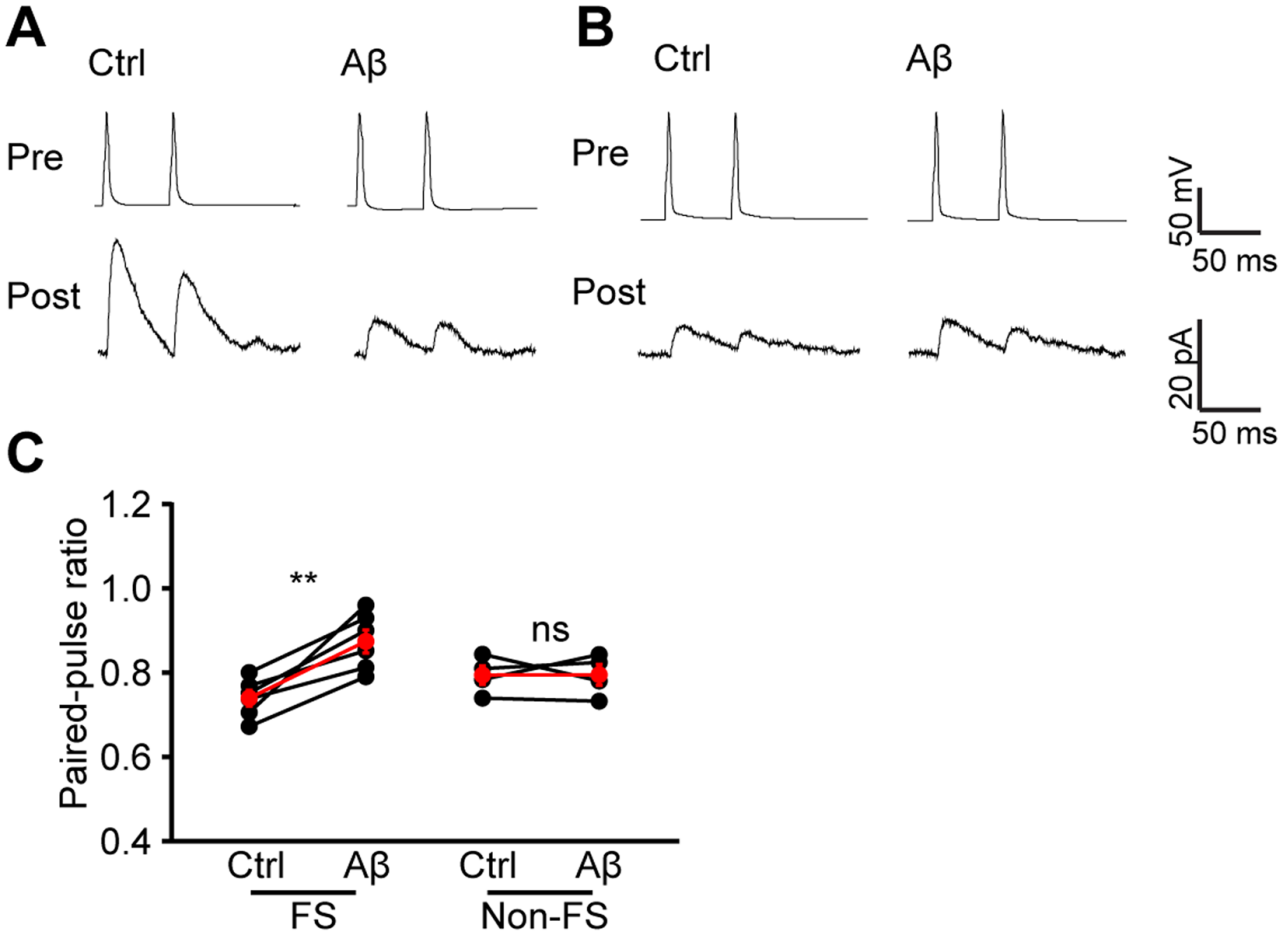
Aβ disrupts GABA release from FS but not non-FS interneurons. (**A**) example traces of uIPSCs triggered by brief paired currents injections with 50 ms interval to the same FS interneuron and recorded in the same pyramidal neuron as in Fig. 3 **A,B** before and after Aβ application; (**B**) example traces of uIPSCs triggered by paired brief current injections with 50 ms interval to the same non-FS interneuron and recorded in the same pyramidal neuron as in Fig. 3 **C,D** before and after Aβ application; (**C**) quantification of PPRs from FS and non-FS interneurons before and after Aβ application. (**p < 0.01).

To examine if presynaptic GABA release from FS interneurons is affected by Aβ application, we monitored the paired pulse ratio (PPR) of uIPSCs. We found that the PPR of FS (Aβ: 0.87 ± 0.03, n = 6 pairs of cells, p = 0.004 compared with Ctrl: 0.74 ± 0.02, paired *t* test; Fig. 4A–C) but not non-FS (Aβ: 0.79 ± 0.02, n = 4 pairs of cells, p = 0.979 compared with Ctrl: 0.79 ± 0.02, paired *t* test; Fig. 4A–C) interneurons was significantly increased after Aβ administration, suggesting that presynaptic GABA release perturbation counts for the disrupted inhibitory input from FS interneurons.

### Excessive activation of D1 receptor is involved in Aβ-induced disruption of inhibitory input and Inhibition of D1 receptor can restore E/I balance

**Figure 5:**
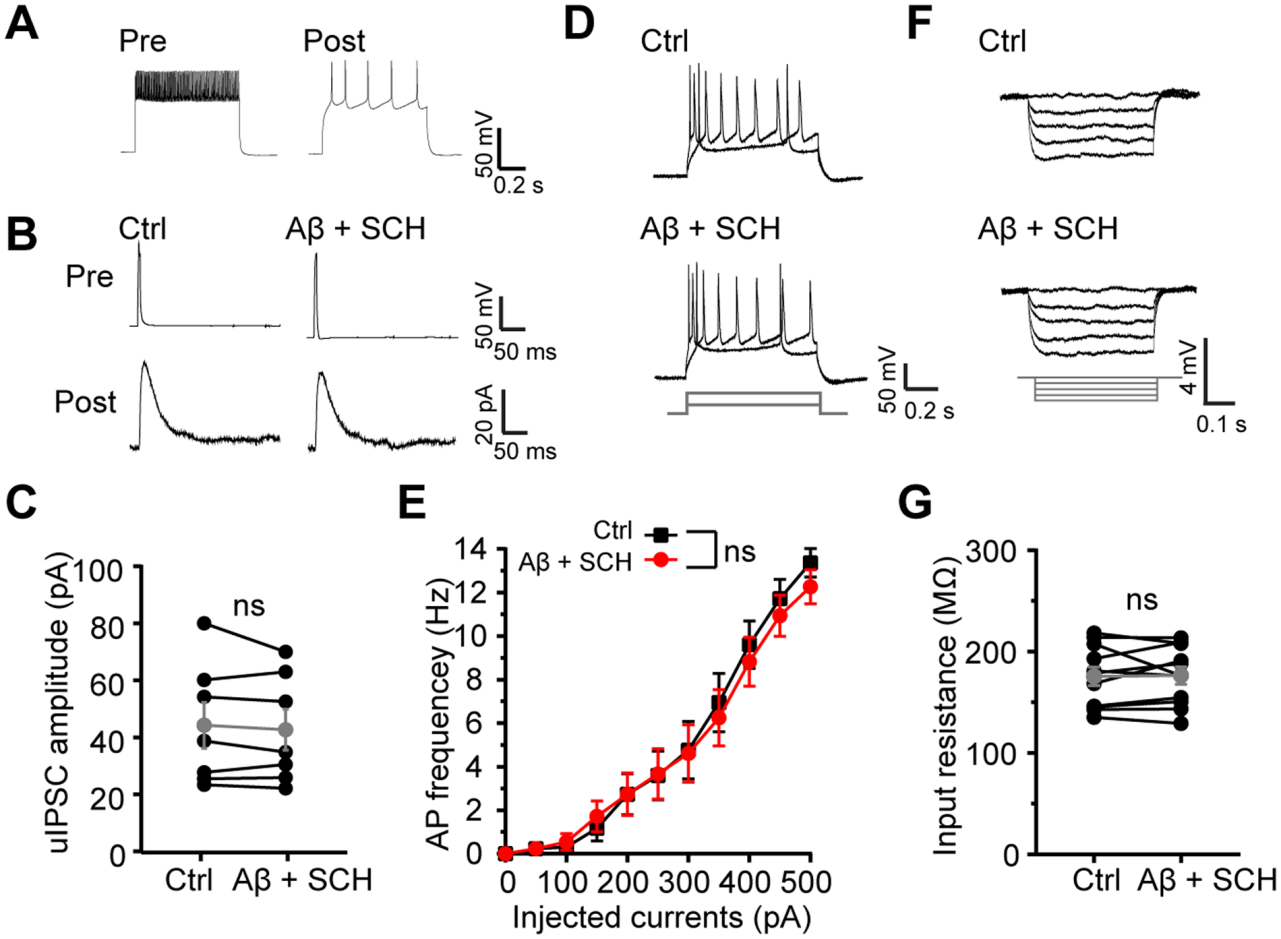
D1 receptor is involved in Aβ-induced disruption of inhibitory input and hyperexcitability of pyramidal cells. (**A**) Example AP traces of a pair of nearby FS interneuron and excitatory neuron simultaneously recorded; (**B**) example traces of uIPSCs triggered by brief current injection to the FS interneuron and recorded in the pyramidal neuron before and after Aβ + SCH23390 application; (**C**) quantification of uIPSCs amplitude from FS interneurons before and after Aβ + SCH23390 application; (**D**) examples of action potential traces induced by 150 pA and 400 pA current injections before and after Aβ + SCH23390 application; (**E**) quantification of the APs frequencies at different current injections before and after Aβ + SCH23390 application; (**F**) examples of membrane potential responses induced by serial currents injection from −20 pA to 0 pA before and after Aβ + SCH23390 application; (**G**) quantification of cellular input resistance before and after Aβ + SCH23390 application.

GABA release from the axonal terminal of FS interneurons is regulated by dopamine through activating D1 receptor^35^. The dopaminergic neurons are related to Aβ-induced pathological processes during early onset of Alzheimer’s disease^44^. Moreover, dopamine release can be promoted by nanomolar Aβ in frontal cortex^38^ and D1 receptor participates in Aβ-induced epileptic activity^39^. To examine if a dopamine-dependent signaling pathway is involved in Aβ-induced inhibitory input disruption and E/I imbalance, we applied 10 µM D1 receptors antagonist SCH23390 (SCH) with Aβ into perfusing ACSF. Interestingly, inhibition of D1 receptor largely ameliorated Aβ-induced disruption of inhibitory input from FS interneurons (Aβ + SCH: 42.65 ± 7.19 pA, n = 7 pairs of cells, p = 0.393 compared with Ctrl: 44.19 ± 8.05 pA, paird *t* test; Fig. 5A–C). More importantly, D1 receptor antagonist also reversed Aβ-induced hyperexcitability of excitatory pyramidal cells (p = 0.1519 compared with Ctrl, repeated-measures two-way ANOVA, n = 10 cells; Fig. 5D,E) and increase of input resistance (Aβ + SCH: 175.62 ± 9.15 MΩ, n = 12 cells, p = 0.8479 compared with Ctrl: 176.48 ± 8.67 MΩ, paird *t* test; Fig. 5F,G). As a control, SCH23390 itself did not change basal excitatory synaptic transmission (sp EPSCs frequency: 4.66 ± 0.54 Hz, n = 4 cells, p = 0.5190 compared with Ctrl: 4.43 ± 0.35 Hz; sp EPSCs amplitude: 6.08 ± 1.09 pA, n = 4 cells, p = 0.5190 compared with Ctrl: 6.78 ± 1.37 pA; paired *t* test; Supplemental Fig. 5). These results suggest that D1 receptor is involved in Aβ-induced disruption of inhibitory input and E/I imbalance.

To further confirm that D1 receptor plays a critical role in Aβ-induced disruption of inhibitory input and E/I imbalance, we applied a D1 receptor agonist to see if activating D1 receptor can mimic and occlude Aβ-induced disruption of inhibitory input from FS interneurons. Indeed, application of the D1 receptor agonist SKF 38393 (SKF) dramatically decreased uIPSCs amplitude to about 47% (47.26%, n = 4 pairs of cells, p = 0.0015 compared with Ctrl, paired *t* test; Supplemental Fig. 6), and application of Aβ after SKF administration failed to further decrease uIPSCs amplitude (44.74%, n = 4 pairs of cells, p = 0.2466 compared with SKF; p = 0.0003 compared with Ctrl; Supplemental Fig. 6), confirming Aβ and D1 receptor function in the same signaling pathway to disrupt inhibitory input and cause E/I imbalance.

**Figure 6:**
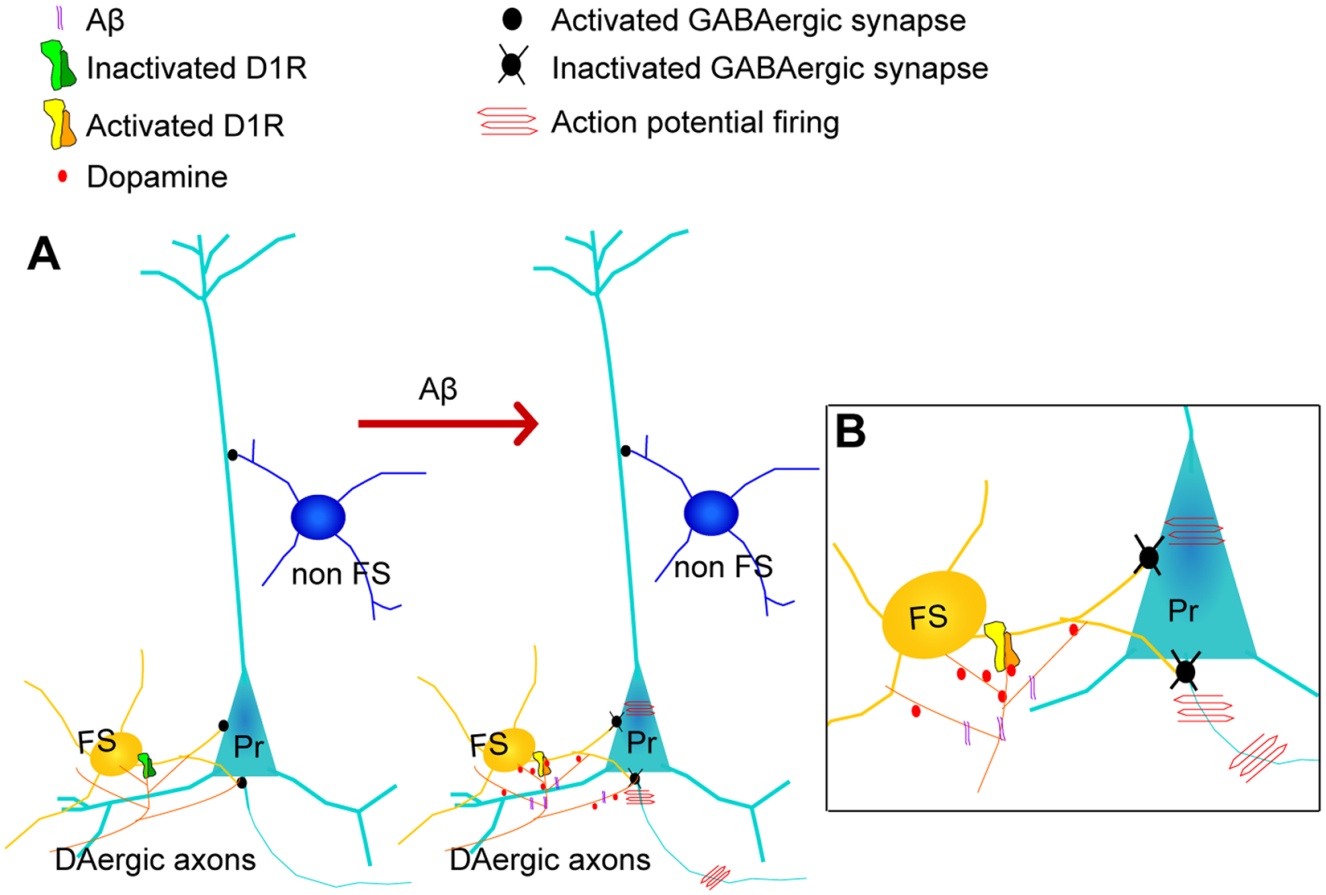
Working model: Aβ induces hyperexcitability of excitatory pyramidal cells through D1 receptor-dependent disruption of GABAergic inhibitory input from FS interneurons. (**A**) In this model based on our results, we propose that low concentration of 50 nM Aβ promotes dopamine release from dopaminergic (DAergic) axons in ACC, dopamine in turn excessively activate D1 receptors on FS interneurons which dramatically inhibit GABA release from FS interneurons. These sequential events ultimately lead to E/I imbalance and hyperexcitability of excitatory pyramidal cells in ACC; (**B**) Zoom-in image shows this process.

Thus, taken together, our results show that Aβ promotes dopamine release from dopaminergic (DAergic) axons in ACC, and the excessive dopamine overactivates D1 receptors of FS interneurons which dramatically inhibits GABA release and then leads to E/I imbalance in ACC (Fig. 6A,B).

## Discussion

The present study demonstrates that 50 nM Aβ leads to hyperexcitability of excitatory pyramidal cells in ACC through specifically disrupting inhibitory input from FS interneurons. Moreover, the perturbation of presynaptic GABA release is the main cause of this inhibitory input disruption. Interestingly, this study also illustrates that excessive dopamine action on D1 receptor on FS interneurons plays a key role in the Aβ-induced perturbation of inhibitory innervation and hyperexcitability of pyramidal cells.

Aβ is progressively accumulated during AD development, and Aβ accumulation-induced disruption of neuronal signaling is the best correlation with neuropathology in AD patients^2–4^. Many studies suggest that Aβ functions in a concentration-dependent manner, low concentrations of Aβ (pM to nM) can induce presynaptic calcium level increases^45^ and also promote neuronal excitability and plasticity^12,13,46^, whereas high concentration of Aβ (µM) depresses excitatory and inhibitory synaptic transmission or plasticity^41,47,48^. Nanomolar Aβ concentrations (1–100 nM) are more likely to aggregate into the small oligomers and fibrils and are believed to be most pathologically relevant in AD patients and in AD animal models^49^. In this study, acute application of 50 nM Aβ on mouse ACC is shown to disrupt E/I balance and lead to hyperexcitability of pyramidal cells.

Many mechanisms underlying neuronal hyperexcitation have been found in *in vitro* Aβ exposure paradigms as well as in Aβ overexpression AD animal models. For example, in cultures of primary mouse hippocampus pyramidal cells, upregulation of α7-nAChRs is necessary for production of chronic Aβ-induced neuronal hyperexcitation^12^. In transgenic Drosophila line that overexpresses a secreted form of the toxic human Aβ_1–42_, selective degradation of the highly conserved A-type K+ channel, Kv4 leads to an increased AP firing and neuronal hyperactivity^13^. In addition, impairment of E/I balance has also been found in APP-overexpressed AD mouse models^50,51^ and in *in vitro* Aβ exposure^50^. Moreover, the decreased level of the interneuron specific PV cell-predominant voltage-gated sodium channel subunit Nav1.1 leads to decreased inhibitory synaptic activity and enhanced hypersynchrony, memory deficits and premature mortality^51^. In our study, we found that disruption of inhibitory input from FS interneurons is responsible for the E/I imbalance caused by acute Aβ exposure in ACC, and perturbation of presynaptic GABA release leads to the inhibitory input disruption and E/I imbalance.

Inhibitory interneurons can be classified into the Ca2+-binding protein parvalbumin (PV), the neuropeptide somatostatin (SST) and the ionotropic serotonin receptor 5HT3a (5HT3aR) subtypes based on neuronal markers, they can also be classified into FS and non-FS subtypes based on AP firing patterns^52^. The PV FS group accounts for ~40% of GABAergic neurons and are the major source of perisomatic inhibition onto excitatory pyramidal cells^32,52^. Our results are in line with previous studies to support that FS interneurons play a pivotal role in Aβ-induced E/I imbalance and hyperexcitability of pyramidal cells.

Enriched dopamine innervation of frontal cortex has been implicated both in the modulation of normal cognitive processes such as working memory formation and in many neurobiological diseases including age-related memory decline and schizophrenia^53^. Dopamine regulates the recurrent excitatory transmission between pyramidal cells^36^, it also depresses inhibitory input from FS interneurons and enhances inhibitory input from non-FS interneurons^35^. Excessive dopamine innervation of ACC FS PV interneurons has been suggested to disrupt E/I balance in schizophrenia^37^. In AD, Aβ can promote dopamine release in frontal cortex through activating α7-nAChRs^38^, and D1 receptor is also involved in Aβ-induced epileptic activity in mice^39^. Here we found that excessive D1 receptor activation is involved in Aβ-induced E/I imbalance in ACC, highlighting pathological roles the dopamine-D1 receptor signaling pathway plays in AD.

ACC plays a pivotal role in memory, attention and emotion^17–19^. ACC also represents one of most vulnerable areas and “epicenters” during the pathological course of AD^23–26^. Here our results illustrate Aβ can cause hyperexcitability of pyramidal cells through D1 receptor-dependent disruption of inhibitory input from FS interneurons in ACC. This indicates that Aβ-induced dysfunction of excitatory, inhibitory and neuromodulatory circuitries in some key areas are the critical pathological mechanisms underlying cognitive decline in AD.

## Methods

All animal experiments in this study were approved by Medical Research Ethics Committee of Nanjing Medical University and performed in accordance with the approved guidelines and regulations.

### Aβ_1–42_ preparation

Aβ _1–42_ and scrambled peptides were purchased from ChinaPeptides Company in Shanghai, China. Soluble peptide solution was prepared as previously^41^. In brief, the peptide was dissolved in dimethyl sulfoxide at a concentration of 10 mM and then diluted 100 times into phosphate-buffered saline (PBS). After that, it was vortexed for 30 min at room temperature and centrifuged 15,000 g at 4 °C for 1 h. The supernatant was immediately aliquoted and stored at −20 °C. Aliquots were diluted into perfusing ACSF to a final concentration of 50 nM.

### Brain slice preparation

Acute Coronal brain slices were prepared as previously^40,41^. Briefly, 2–3 weeks old CD1male mice were deeply anesthetized with avertin and decapitated. The brains were immediately removed and cut into 350 μm coronal slices using a vibrating blade microtome in ice-cold ACSF containing (in mM) 126 NaCl, 3 KCl, 1.25 KH2PO4, 1.3 MgSO4, 3.2 CaCl2, 26 NaHCO3 and 10 glucose, bubbled continuously with Carbogen (95% O2/ 5% CO2) at pH 7.4. Fresh slices were incubated in a chamber with continuously carbo-generated ACSF at 34 °C and recovered for at least 1 h before recording.

### Whole-cell electrophysiological recording and immunostaining and confocal scanning

For recording of APs from pyramidal cells, whole-cell recordings in the voltage-clamp mode were made as previously^40,41^ with patch pipettes containing (in mM): 130 potassium-gluconate, 6 KCl, 2 MgCl2, 0.2 EGTA, 10 HEPES, 2.5 Na2ATP, 0.5 Na2GTP, 10 potassium-phosphocreatine and 0.5% neurobiotin for morphological reconstruction (Vector Lab) (pH 7.25 and 295 mOsm kg^−1^). 800 ms depolarizing current steps from −20 pA to 500 pA with 5pA interval were injected to induce serial APs to study neuronal intrinsic properties like cellular excitability and input resistance before and 5–10 mins after Aβ administration into perfusing ACSF. Input resistance was calculated as 800 ms −10 pA current injection-evoked steady membrane potential divided by the injected current. Mini IPSCs were recorded with patch pipettes containing (in mM): 135 KCl, 2 MgCl2, 0.1 EGTA, 10 HEPES, 2 Na2ATP, 0.2 Na2GTP with 1 μM TTX and AMPA receptor antagonist NBQX (10 μM, Tocris) in perfusing ACSF. For recording of uIPSCs from interneurons to pyramidal cells, multiple-channels patches of at least 1 interneuron and 1–3 pyramidal cells were made and whole cell recordings were formed with internal solution (in mM): 130 mM potassium-gluconate, 6 KCl, 2 MgCl2, 0.2 EGTA, 10 HEPES, 2.5 Na2ATP, 0.5 Na2GTP, 10 potassium-phosphocreatine and 0.5% neurobiotin. A brief of current (5ms) was injected to induce single AP in interneurons under current clamping configuration and uIPSCs were simultaneously recorded from postsynaptic pyramidal cells with membrane potential clamped around EPSC reversal potential 0–10 mV. Paired-pulse ratio (PPR) of uIPSCs was recorded with paired-currents injections with 50 ms inter-pulse interval. The PPR was calculated as the ratio of the second IPSC amplitude to the first. 15 sweeps of paired EPSCs before and 5–10 mins after Aβ application were averaged and calculated separately. Synaptic responses collected with an Axopatch-700B amplifier (Molecular Devices, Palo Alto, CA), filtered at 2 kHz and digitized at 5–10 kHz. Data were analyzed using Clampfit 9.2 (Molecular Devices, Palo Alto, CA).

Some brain slices after recording were fixed in 4% formaldehyde overnight and stained with nuclear dye DAPI and streptavidin (Invitrogen) for visualizing recorded cells. Images were scanned by Olympus FV1000 confocal microscope.

### Drugs

SCH23390, SKF 38393 and BMI were purchased from Tocris. NBQX were purchased from Sigma-Aldrich. These agents were prepared in either distilled water or DMSO and immediately stored in aliquots at −20 °C. The aliquot was diluted directly in the perfusing ASCF during experiments.

### Statistical analysis

All values were described as mean ± SEM. Statistical significance was assessed using repeated-measures two-way ANOVA, paired-sample *t* test, independent-sample *t* test (two populations) or Wilcoxon signed-rank test . Differences were considered significant when p ≤ 0.05 (*0.05, **0.01,***p < 0.001).

### Data availability

The datasets generated during and/or analyzed during the current study are available from the corresponding author on reasonable request.

## Electronic supplementary material

Supplementary figures
