# Supplementary material for: Amyloid β causes excitation/inhibition imbalance through dopamine receptor 1-dependent disruption of fast-spiking GABAergic input in anterior cingulate cortex
Source: Sci Rep. 2018 Jan 10;8:302. doi: 10.1038/s41598-017-18729-5 (PMC5762926; doi:10.1038/s41598-017-18729-5)
Supplement: Supplementary file 1 — Supplementary figures [file 41598_2017_18729_MOESM1_ESM.pdf]

Amyloid β causes excitation/inhibition imbalance through dopamine receptor 1-dependent disruption of fast-spiking GABAergic input in anterior cingulate cortex

Si-Qiang Ren[1, 4, 5], Wen Yao[2, 4], Jing-Zhi Yan[3], Chunhui Jin[1], Jia-Jun Yin[1], Jianmin Yuan[1], Shui Yu[1], Zaohuo Cheng[1, 5]

[1] Wuxi Mental Health Center, Nanjing Medical University, Wuxi China.

[2] Department of Pharmacology, Wuxi Higher Health Vocational Technology School, Wuxi China.

[3] Jiangsu Key Laboratory of Brain Disease Bioinformation, Research Center for Biochemistry and Molecular Biology, Xuzhou Medical College, Xuzhou China.

[4] Equal contribution.

[5] Correspondence should be addressed to S.R. (email: rensiqiang159@126.com) or Z.C. (email: chengzaohuo@126.com)

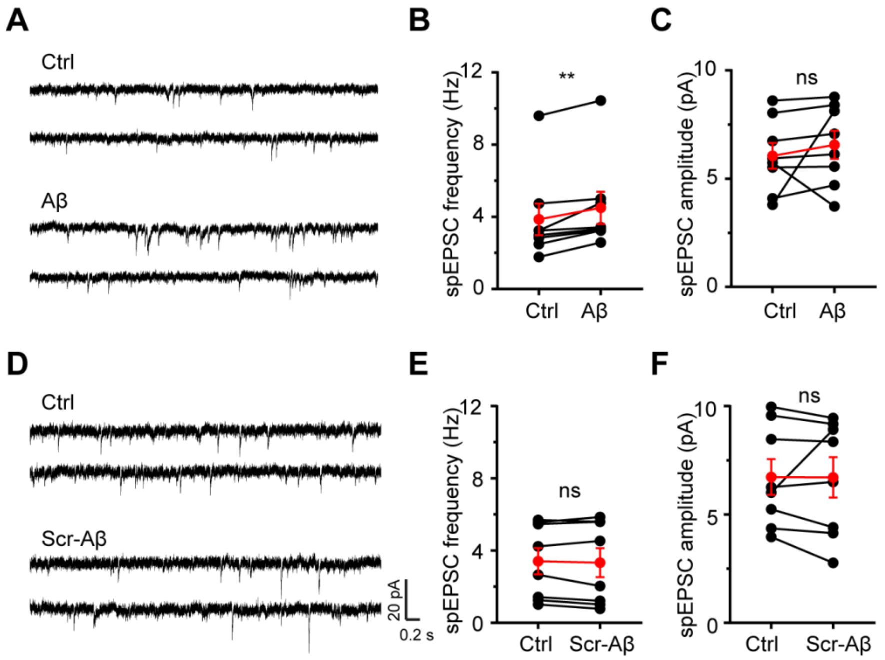

Supplemental Fig. 1 Aβ induces hyperexcitation of excitatory pyramidal cells in ACC. A, example traces of sp EPSCs of excitatory pyramidal cells in ACC before and after Aβ application; **B**, quantification of sp EPSCs frequency before and after Aβ application; **C**, quantification of sp EPSCs amplitude before and after Aβ application; **D**, example traces of sp EPSCs of excitatory pyramidal cells in ACC before and after Scr-Aβ application; **E**, quantification of sp EPSCs frequency before and after Scr-Aβ application; **F**, quantification of sp EPSCs amplitude before and after Scr-Aβ application. (**$p < 0.01$).

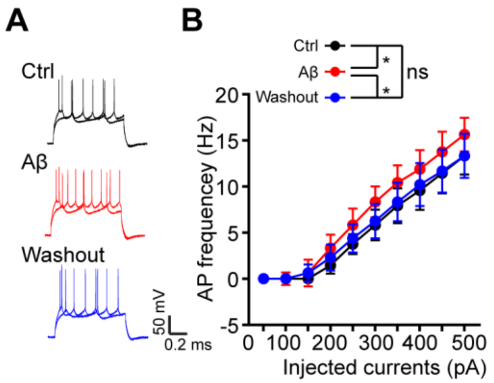

Supplemental Fig. 2 Aβ-induced hyperexcitation of excitatory pyramidal cells can be reversed after Aβ washout. A, examples of AP traces before, during Aβ exposure and after its washout; **B**, quantification of the AP frequencies at different current injections before, during Aβ exposure and after its washout. (*$p < 0.05$).

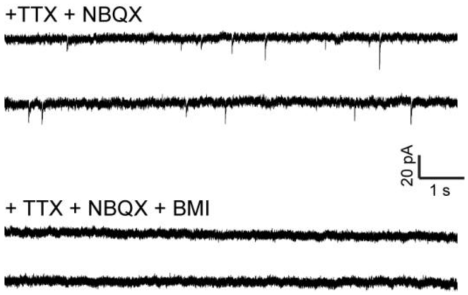

Supplemental Fig. 3 Isolation of mini IPSCs. Robust mini PSCs isolated by adding TTX and NBQX with high chloride internal solution are totally blocked by $GABA_A$ receptor inhibitor BMI, suggesting the isolated mini PSCs are pure mini IPSCs.

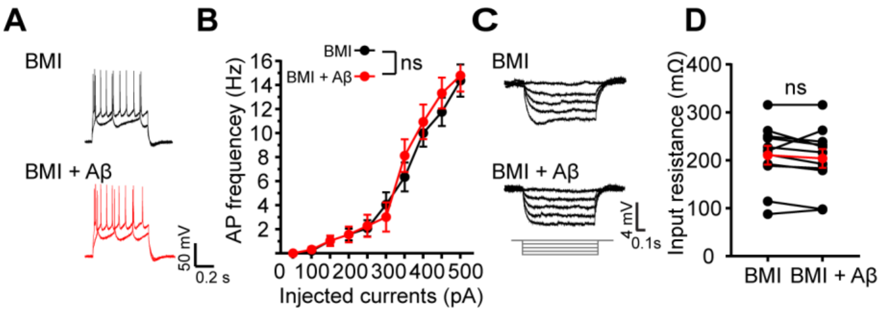

Supplemental Fig. 4 Aβ-induced hyperexcitation of excitatory pyramidal cells can be blocked by inhibiting GABAergic inhibitory input. A, examples of AP traces in BMI and BMI + Aβ conditions; **B**, quantification of the AP frequencies at different current injections in BMI and BMI + Aβ conditions; **C**, examples of membrane potential responses induced by serial currents injection from -20 pA to 0 pA with 5 pA interval in BMI and BMI + Aβ conditions; **D**, quantification of cellular input resistance in BMI and BMI + Aβ conditions.

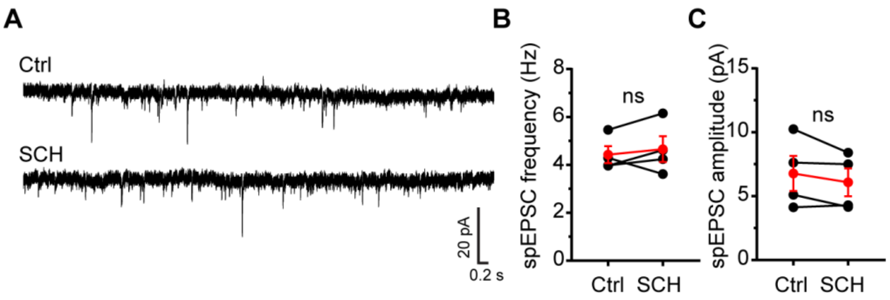

Supplemental Fig. 5 SCH 23390 itself does not affect basal excitatory synaptic transmission. A, example traces of sp EPSCs of excitatory pyramidal cells in ACC before and after SCH23390 application; **B**, quantification of sp EPSCs frequency before and after SCH23390 application; **C**, quantification of sp EPSCs amplitude before and after SCH23390 application.

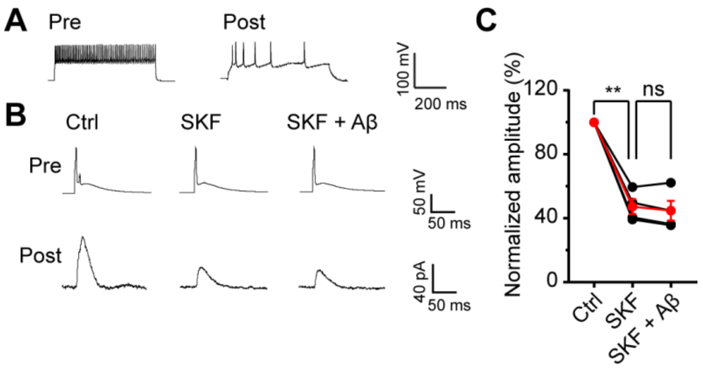

Supplemental Fig. 6 D1 receptor agonist SKF 38393 can mimic and occlude Aβ-induced disruption of inhibitory input from FS interneurons. A, example AP traces of a pair of nearby FS interneuron and excitatory neuron simultaneously recorded; **B**, example traces of uIPSCs triggered by brief current injection to the FS interneuron and recorded in the pyramidal neuron in different conditions; **C**, quantification of normalized uIPSCs amplitude in different conditions. (**$p < 0.01$).

;
